# Complications, treatment, and follow-up of peutz-jeghers syndrome: About 2 case reports

**DOI:** 10.1016/j.ijscr.2023.108511

**Published:** 2023-07-19

**Authors:** Yacine Ouadi, Maryem Ben Brahim, Emna Trigui, Wassim Frikha, Fadhel Fterich, Montasser Jameleddine Kacem

**Affiliations:** a Department of Surgery A La Rabta Hospital, Tunis, Tunisia; b Faculty of Medicine of Tunis, Tunis El Manar University, Tunis, Tunisia; c Department of Radiology La Rabta hospital, Tunis, Tunisia

**Keywords:** Peutz jeghers polyposis, Gastrointestinal intussusception, Surgical polypectomy, Case report, General surgery

## Abstract

**Introduction:**

Peutz-Jeghers syndrome is an inherited disorder distinguished by hamartomatous polyps in the gastrointestinal tract and pigmented mucocutaneous lesions.

Treatment of the polyps is never definitive, with most patients needing several laparotomies. For this reason, surgeons should be economical in terms of surgical resection to prevent a short bowel syndrome in the long run.

In this paper, we report two observations of patients presented a Peutz Jeghers syndrome (PJS).

**Cases presentation:**

Case report 1: A 32-year-old women, who was operated on for an *intestinal perforation related to a Peutz-jeghers hamartoma of the small bowel and was later re operated on for* colonic intussusception,

Case report 2: A 15-year-old patient that has been operated on three times already for small bowel intussusception and later for duodenal obstruction.

**Clinical Discussion:**

In an attempt to reduce complications, the 2010 guidelines updated in 2021 by the European Hereditary Tumor group introduced obligatory monitoring by fibroscopy and colonoscopy associated with an entero-MRI or a videocapsule from the age of 8 years.

Laparotomy is indicated when endoscopic treatment is impossible or in emergency setting. When surgery is indicated, intestinal resection should be reserved for rare cases in order to avoid short bowel syndrome.

The association of an intraoperative endoscopic treatment is recommended by some authors.

**Conclusion:**

Peutz Jeghers syndrome is a rare entity with a complicated surveillance. Adequate polyp mapping is necessary for adequate planning of the treatment. The need for multiple laparotomies makes a comprehensive approach to surgery mandatory to prevent short bowel syndrome.

## Highlights

• Peutz Jeghers syndrome is a rare entity with a demanding surveillance.
• Adequate polyp mapping is necessary for adequate planning of the treatment.
• The need for multiple laparotomies during evolution of the disease makes the surgical approach based on limited resection to prevent short bowel syndrome.

## 1 Introduction

Peutz-Jeghers syndrome is an inherited, autosomal dominant disorder distinguished by hamartomatous polyps in the gastrointestinal tract and pigmented mucocutaneous lesions.

Most patients with Peutz-Jeghers syndrome are diagnosed in the second or third decade of life by a complication of the polyps such as hemorrhage, anemia, intussusception or acute intestinal obstruction [1].

Treatment of the polyps is never definitive, with most patients needing several laparotomies. For this reason, surgeons should be economical in terms of surgical resection to prevent a short bowel syndrome in the long run [2].

In this paper, we report two observations of patients presented a PeutzJeghers syndrome (PJS). Through these two cases, we describe the clinical characteristics, complications, and treatment of this disease.

This case report has been reported in line with the SCARE Criteria [3].

## 2 Case presentation

### 2.1 Case report 1

A 32-year-old, asthmatic patient, with a history of a Peutzjeghers syndrome since 2004 confirmed by pigmented mucocutaneous lesions associated with colonic hamartomas confirmed by pathology.

The patient was operated on in 2012 in our department for a peritonitis secondary to jejunal volvulus upstream of a large polyp located at 80 cm from the Treitz angle. She had a 20-cm resection of the small intestine with ileostomy. The patient had severe malnutrition and ionic disorders post-operatively, leading to an early stoma reversal.

Currently, she is being managed for recurrent intestinal obstruction related to colonic intussusceptions.

She was explored by a CT scan which showed a polyp of the splenic flexure responsible for Colo-colic intussusception. There were other uncomplicated and small colonic polyps distributed. Colonoscopy showed a 4cmpolyp with a large pedicle at the level of the splenic flexure and many other polyps with hyperplasic appearance throughout the colon.

**Fig. 1:**
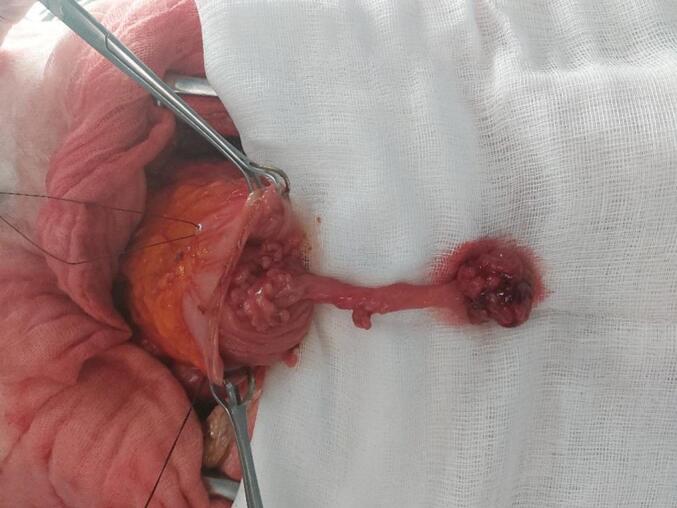
A polyp of 4 cm diameter which has a long pedicle of 10 cm with multiple small sessile polyps near his implantation base.

We concluded that the splenic flexure polyp is responsible for the symptoms. And since endoscopic resection was not possible due the to the large pedicle, we decided to perform a laparotomy to remove the polyp. We performed a median laparotomy and we found many centimetric polyps in the small intestine and a unique right transverse polyp of 2 cm in diameter. A colotomy was performed and it revealed a polyp of 4 cm diameter with a long 10 cm pedicle. There were also multiple small sessile polyps near his implantation base (Fig. 1).

Given the symptomatic nature of the treatment and the benign nature of polyps, we decided to perform a polypectomy rather than segmental colectomy. This was obtained safely by a linear stapler. The postoperative course was uneventful. Pathology examination showed an hamartoma without any malignancy. The patient did not have any symptoms at one year follow up.

### 2.2 Case report 2

We report the case of a 15-year-old patient, with no family history of polyposis, that was diagnosed with Peutz-Jeghers syndrome. This diagnosis was confirmed when the patient was operated for a small intestine intussusception related to 2 centimetric polyps at 4-year-old. He had a resection of 15 cm of the small intestine removing the 2 polyps with side-to-side enteric anastomosis. Pathology showed a hamartoma and genetic study found the STK1 mutation.

In 2015 and 2016, he was operated for small bowel intussusceptions. Polypectomies through small enterotomies were performed. The patient remained asymptomatic since 2016.

**Fig. 2:**
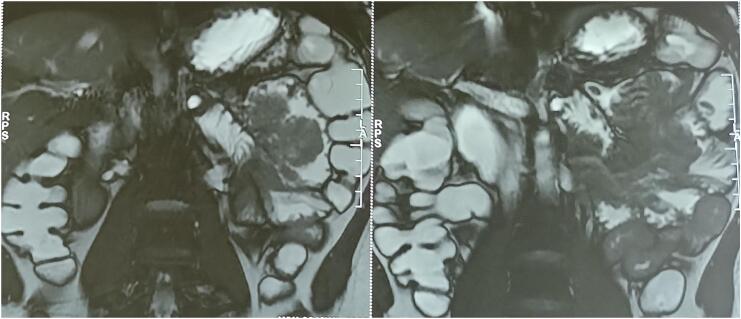
Entero-MRI showing a jejuno-jejunal intussusception associated with a voluminous duodenal polyp,7 cm wide with a large pedicle.

**Fig. 3:**
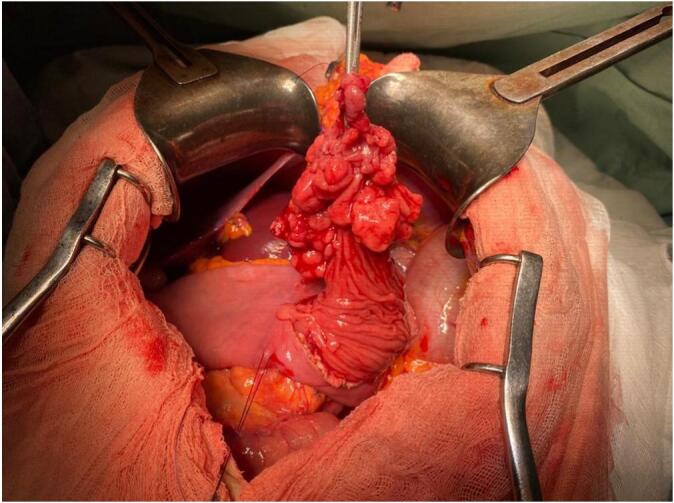
Large polypoid mass measuring approximately 7 cm located in the first jejunal loop.

In April 2022, an entero-MRI performed as part of his regular monitoring showed the presence of a jejuno-jejunal intussusception associated with a voluminous duodenal polyp,7 cm wide with a large pedicle (Fig. 2). Upper endoscopy showed numerous small hyperplasic polyps in the stomach and a circumferential voluminous polypoid formation extended over 9 cm taking the entire duodenum D2 and D3. This formation remains at a distance from the duodenal papilla. The mucosa of the first jejunal loop was normal. Colonoscopy showed no abnormalities. Endoscopic resection was not possible due to the wide implantation base of the polyp. We decided to operate the patient. Through a midline laparotomy we found a jejuno-jejunal intussusception upstream of a large polypoid mass measuring approximately 7 cm located in the first jejunal loop (Fig. 3). The palpation of the rest of the small bowel including the duodenum did not find other polyps.

In fact, the polyp of the jejunum was very mobile due to a long pedicle and was responsible of the intussusception. A jejunotomy was performed and the polypoid mass was removed by a linear stapler. The pathological examination of the specimen confirmed the hamartomatous nature of this polyp, without signs of malignant transformation. The early postoperative course was marked by an acute intestinal intussusception related to a distal polyp in the ileum. Medical treatment allowed avoiding a reoperation and the patient was discharged with normal transit. The patient did not have any symptoms at 6 months follow up.

## 3 Discussion

Peutz-Jeghers syndrome (PJS) is an autosomal dominant condition characterized by hamartomatous gastrointestinal polyps and mucocutaneous pigmentation. It is a rare entity with a prevalence of around 1 in 100.000 people which is why we thought it would be interesting to see how we managed two cases in our department to shed further light on this disease.

This syndrome has no racial nor sex predominance and is usually diagnosed during the first two decades of life [4]. This was the case for both our patients.

The diagnosis made at a young age in presence of the periorificial lentiginosis in our two patients. The diagnosis did not avoid complications despite monitoring based on the clinic alone. In an attempt to avoid complications, the 2010 guidelines updated in 2021 by the European Hereditary Tumor group introduced obligatory monitoring by fibroscopy and colonoscopy associated with an entero-MRI or a videocapsule from the age of 8 years [1,5].

This monitoring scheme is not the only one, Xu in a large series of 566 patients suggested monitoring by imaging (ultrasound, MRI, video-capsule) before the age of 10 and combines complete upper and lower endoscopic exploration from 10 years [6]. Indeed, polyp related symptoms usually arise by the age of 10 in 33 % of patients and by the age of 20 in 50 % of them [1].

These surveillance schemes are not easy to follow due to their expensive, invasive and demanding nature especially in low-income countries. However, the majority of authors agree that the discovery of a polyp greater than or equal to one cm authorizes its endoscopic resection [1,2,6]. The European hereditary tumor group 2021 guidelines indicates endoscopic resection from 1.5 cm [5]. This limit of 1.5 cm is also adopted by the European Society of Gastrointestinal Endoscopy (ESGE) and European Society for Pediatric Gastroenterology Hepatology and Nutrition (ESPHAN) [5].

This monitoring should not omit the risk of degeneration of the polyps which is evaluated between 2 and 10 % [1,6,7]. This risk increases with age and reaches 70 % by 60 years old [6]. Increased risk for extraintestinal cancer of the pancreas, gallbladder, common bile duct, breast, and thyroid is also reported [1,6,7].

Patients with Peutz Geghers are potentially surgical during the course of their disease. In fact, for Xu et al., 70 % of patients underwent laparotomy. This laparotomy was iterative in our two patients and around 30 % for Xu et al., with a maximum of 7 surgeries for one patient [6].

This laparotomy most of the time concerns the small intestine and the colon, given the frequent distribution of these polyps in those segments. Indeed, polyps are most commonly found in the small intestine (60–90 %) and colon (50–64 %) [1]. These operations were carried out urgently for our two patients and intussusception is the most frequent complication [8,9].

Moreover, the particularity of polyposis in the context of the peutz jeghers syndrome is that the recurrence of symptoms and complications is certain. Bowel sparing philosophy is mandatory.

Laparotomy is indicated only when endoscopic treatment is impossible or if there is an emergency setting. When surgery is indicated, polypectomy should be performed through entorotomy, while intestinal resection should be reserved for rare cases in order to avoid short bowel syndrome in the long run.

Better still, the association of an intraoperative endoscopic treatment in presence of adequate equipment and a trained endoscopist, makes it possible to eradicate in a preventive manner most polyps as recommended by some authors [1,2,5,6].

## 4 Conclusion

Peutz Jeghers syndrome is a rare entity with a demanding surveillance. Adequate polyp mapping is necessary for adequate planning of the treatment. The need for multiple laparotomies during evolution of the disease makes the surgical approach based on limited resection to prevent short bowel syndrome.

## CRediT authorship contribution statement

Yacine Ouadi, Conceptualisation, Redaction, Data curation, Project administration.

Maryem Ben Brahom, Conceptualisation, Redaction, Data Curation, Project Administration.

Emna Trigui Conceptualisation, Redaction,

Wassim Frikha Photography Rendering, Data Curation.

Fadhel Fterich Supervision, Validation, Visualisation.

Montasser Jameleddine Kacem Supervision, Validation, Visualisation.

## Sources of funding

No sources of funding.

## Ethical approval

The article is a case report that does not require ethical approval in our country to be published, only patient consent is necessary. The study is exempt from ethnical approval in our institution.

## Consent

Written informed consent was obtained from the patient for publication of this case report and any accompanying images. A copy of the written consent is available for review by the Editor-in-Chief of this journal on request. Written informed consent was obtained from the patient's parents/legal guardian for publication and any accompanying images. A copy of the written consent is available for review by the Editor-in-Chief of this journal on request.

## Provenance and per review

Not commissioned, externally pee-reviewed.

## Research registration

Not applicable.

## Guarantor

Yacine Ouadi.

## Declaration of competing interest

All authors declare they have no conflict of interest.
